# Endothelial Robo4 suppresses endothelial-to-mesenchymal transition induced by irradiation and improves hematopoietic reconstitution

**DOI:** 10.1038/s41419-024-06546-4

**Published:** 2024-02-21

**Authors:** Seyram Yao Adzraku, Can Cao, Qi Zhou, Ke Yuan, Xiaowen Hao, Yue Li, Shengnan Yuan, Yujin Huang, Kailin Xu, Jianlin Qiao, Wen Ju, Lingyu Zeng

**Affiliations:** 1 Blood Diseases Institute, Xuzhou Medical University, Xuzhou, 221002 China; 2 Key Laboratory of Bone Marrow Stem Cells, Jiangsu Province, Xuzhou, 221002 China; 3 Department of Hematology, Affiliated Hospital of Xuzhou Medical University, Xuzhou, 221002 China; 4 Xuzhou Ruihu Health Management Consulting Co, Ltd, xuzhou, 221002 China

**Keywords:** Extracellular matrix, Stem-cell niche

## Abstract

Bone marrow ablation is routinely performed before hematopoietic stem cell transplantation (HSCT). Hematopoietic stem and progenitor cells (HSPCs) require a stable bone marrow microenvironment to expand and refill the peripheral blood cell pool after ablation. Roundabout guidance receptor 4 (Robo4) is a transmembrane protein exclusive to endothelial cells and is vital in preserving vascular integrity. Hence, the hypothesis is that Robo4 maintains the integrity of bone marrow endothelial cells following radiotherapy. We created an endothelial cell injury model with γ-radiation before Robo4 gene manipulation using lentiviral-mediated RNAi and gene overexpression techniques. We demonstrate that Robo4 and specific mesenchymal proteins (Fibronectin, Vimentin, αSma, and S100A4) are upregulated in endothelial cells exposed to irradiation (IR). We found that Robo4 depletion increases the expression of endoglin (CD105), an auxiliary receptor for the transforming growth factor (TGF-β) family of proteins, and promotes endothelial-to-mesenchymal transition (End-MT) through activation of both the canonical (Smad) and non-canonical (AKT/NF-κB) signaling pathways to facilitate Snail1 activation and its nuclear translocation. Endothelial Robo4 overexpression stimulates the expression of immunoglobulin-like adhesion molecules (ICAM-1 and VCAM-1) and alleviates irradiation-induced End-MT. Our coculture model showed that transcriptional downregulation of endothelial Robo4 reduces HSPC proliferation and increases HSC quiescence and apoptosis. However, Robo4 overexpression mitigated the damaged endothelium’s suppressive effects on HSC proliferation and differentiation. These findings indicate that by controlling End-MT, Robo4 preserves microvascular integrity after radiation preconditioning, protects endothelial function, and lessens the inhibitory effect of damaged endothelium on hematopoietic reconstitution.

## Introduction

Ablation of the bone marrow (BM) before hematopoietic stem cell transplantation (HSCT) is commonly used to treat a wide array of blood malignancies [1]. Following a BM transplant, hematopoietic stem and progenitor cells (HSPCs) require a stable BM microenvironment to repopulate. Signals from the BM niche are essential for hematopoietic homeostasis [2].

Total body irradiation (IR) used to deplete resident HSCs or leukemic cells and make space available in the BM microenvironment destroys not only endogenous HSCs but also non-hematopoietic cells in the vascular niche [3], which mainly comprises endothelial cells (ECs) [4]. BM ECs lining the interior of blood vessels produce various factors, such as CXCL12 and SCF, to regulate HSC activities in stable or stressful conditions [5]. IR causes endothelial inflammation and hyperplasia. Endothelial dysfunction reduces the cellular capacity to maintain homeostasis [6]. While IR preconditioning enables engraftment and creates the available niche for acute regeneration, many hematopoietic conditions due to changes in normal HSC function, such as BM failure and poor graft function (PGF), develop gradually [7]. BM fibrosis promotes the delay or unsuccessful engraftment [8], and IR causes grade BM fibrosis [9]. One serious complication of radiotherapy is fibrotic reactions, which require intense investigation to understand the intimate mechanisms involved in tissue fibrosis and dysfunction. This study examined the potential role of endothelial-to-mesenchymal transition (End-MT) in the radiation-induced fibrotic process.

In a steady state, ECs are incisively maintained but are capable of exhibiting phenotypic plasticity, including their ability to undergo cellular transdifferentiation, referred to as End-MT [10]. During this process, ECs lose their specific markers and progressively acquire mesenchymal phenotype comprising a stellate-shaped morphology, downregulated cell-cell junctions, and increased cellular motility [11]. At the molecular level, End-MT leads to elevated production of mesenchymal cell-specific markers, including αSma, fibronectin, vimentin, S100A4, etc., and progressive reduction and eventual loss of EC-specific proteins such as VE-Cadherin, PECAM-1, and vWF, etc. [12].

Several studies have unanimously demonstrated that the TGF-β isoforms play a potential role in End-MT induction [13, 14]. Upon binding TGF-β ligands to their receptors, they recruit and phosphorylate several cytoplasmic molecules, most notably the Smad2/3 and Smad1/5 proteins, which bind with Smad4 protein to form a complex [15, 16]. The resulting transcription factor heterocomplex translocates into the nucleus, interacting with other transcription factors such as Snail, Snug, Twist, and Zeb1 to regulate gene expression in the End-MT [17, 18]. TGF-β isoforms activate Smad-independent End-MT pathways through PI3K/Akt/mTOR, P38-MAPK, or ERK1/2 pathways [19, 20]. End-MT is also driven by oxidative stress and hypoxia [21]. These signaling pathways have been shown to promote increased expression of the transcription factor, Snail. Snail1 is one of the principal transcription factors involved in cellular plasticity and inhibiting cell adhesion [22].

Roundabout guidance receptor 4 (Robo4) is another EC-specific protein identified using bioinformatic data mining [23]. Robo4 is localized in both the plasma membrane and the cytoplasm. The activation of Robo4 by the axonal guidance factor Slit2 inhibits the proliferation and migration of retinal microvascular EC and promotes vascular permeability and angiogenesis [24]. Robo4 maintains the integrity of the vasculature in mice by interacting with the receptor of nerve guidance factor netrin, UNC5B [25]. Robo4 is reported to inhibit the activation of Src family kinases involved in the modulation of EC functions [26]. VEGF can negatively regulate Robo4 in microvascular ECs [27].

Under oxidative and osmotic stress, Robo4 expression is regulated by hypoxia-inducible factor-1a (HIF-1a) and specificity protein 1 (SP1) ECs [28, 29]. Our previous study found that Robo4 inhibits the IR-induced permeability of ECs by regulating the junctions [30]. However, not much is known about the role of Robo4 in IR-induced End-MT.

Few studies reported the presence of Robo4, specifically in murine BM ECs and HSPCs, regulating HSC trafficking across the microvessels [31]. Furthermore, Slit2/Robo4 signaling plays a crucial role in HSC homeostasis in the BM niche [32]. This study will investigate the importance of Robo4 in maintaining the microvascular integrity of BM ECs after IR and its subsequent effects on HSC’s function.

## Materials and methods

### Cell culture

Murine microvascular ECs (BEND3 cells, ATCC^®^ CRL-2299™) were grown for a maximum of ten passages in Dulbecco’s Modified Eagle Medium (DMEM, Gibco, catalog number: C11995500BT) supplemented with 10% fetal bovine serum (FBS, Gibco, catalog number: 10099–141) and appropriate concentration of penicillin/streptomycin (Gibco; Thermo Fisher Scientific Inc., Waltham, MA, USA) in a humidified 5% CO_2_ incubator at 37 °C.

### Irradiation treatment

After achieving 80% confluence, microvascular ECs were irradiated with gamma (γ) radiation using a GSR C1 137Cs gamma irradiator (Gamma-Service Medical, Bautzner, Germany) at a dose of 15 Gy (dose rate of 1.88 Gy/min). Until further examination, the irradiated EC cells were grown in humidified 5% CO_2_, 37 °C incubators. Control samples that were not irradiated were processed similarly (culture medium, transport to the accelerator, and incubation conditions).

### Plasmids preparation

PCR amplification of mouse Robo4 was performed with primers incorporating Age 1 (CCGG) and EcoR 1 (AATTCAAAAA) restriction sites (Invitrogen, Carlsbad, USA). The synthesized mRobo4 oligonucleotides: 5′-GCTGACTGTGTCTTCACTGAT CTCGAG ATCAGTGAAGACACAGTCAGC-3′ (Robo4-KD#1), 5′-GCCAACAACCTATGGCTATAT CTCGAG ATATAGCCATAGGTTGTTGGC-3′ (Robo4-KD#2) and 5′-GCCACCAACAATGCTGGGCAA CTCGAG TTGCCCAGCATTGTTGGTGGC-3′ (Robo4-KD#3) with or without the 3′-UTR (TTTTTG) were inserted and cloned into GV248 vector (hU6-MCS-Ubiquitin-EGFP-IRES-puromycin), (Genechem, Shanghai, China) according to Genechem standard procedures and validated by restriction digestion and DNA sequencing. As a transfection control (CON.), we utilized a plasmid bearing a control sequence that did not target any particular gene (TTCTCCGAACGTGT CACGT). Also, Genechem supplied lentiviral vectors harboring Robo4 overexpression vectors (Robo4-OE) and its control vector (CONT). The packaging cell line HEK293T cells were co-transfected with the recombinant GV248 vector plasmid (20 μg) containing the shRobo4 and Robo4-OE vectors, 15 μg pHelper 1.0 vector plasmid (pGAG-POL), and 10 μg pHelper 2.0 vector plasmid (pCMV-VSVG). Two days after transfection, viral supernatant from 293 T cells was filtered through a 0.45 μm syringe filter (Thermo Fisher) and ultracentrifuged (Beckman).

### Stable establishment of Robo4 knockdown and overexpression in microvascular ECs

Overnight, 60 mm culture dishes were seeded with 3 × 10^5^ microvascular ECs and allowed to reach 60–70% confluence. Adherent ECs were transduced with Robo4-KD, Robo4-OE, and control vectors using 1× HitransG P (Genechem, Shanghai, China) in 10% FBS DMEM. GFP positivity and transduction levels were evaluated by fluorescence microscopy and FACS analyses after 72 hours of selecting infected lentiviral cells with 1 µg/ml puromycin (Genechem, Shanghai, China). Quantitative real-time PCR (qRT-PCR) and western blotting were used to analyze changes in target gene expression.

### Total RNA extraction and quantitative reverse transcription-polymerase chain reaction (qRT-PCR)

Trizol reagent (Life Science, Carlsbad, CA, USA) extracted total RNA from a monolayer of adherent ECs after IR. Once the OD 260/280 was measured to be between 1.8 and 2.0 using a NanoDrop 2000c spectrophotometer (NanoDrop Technologies, Rockland, DE, USA), 1.0 µg RNA was used for reverse transcription utilizing Primer Script reverse transcriptase master mix (Takara Biotechnology, Dalian, China). qRT-PCR was performed in triplicate in 96-well plates. Each well had 2.0 μL of cDNA, 200 nM forward primer, 200 nM reverse primer, 1x SYBR Green I Master mix, and up to 20 μL of DNase/RNase-free distilled water. The 96-well plates were placed in a Roche Lightcycler 480 II (Life Sciences, Roche) for real-time PCR analysis. This PCR cycling program was used: 10 minutes at 95 °C, 40-cycles at 95 °C for 30 seconds, 30 seconds at 60 °C and 15 seconds at 72 °C; 15 seconds at 95 °C using the Light Cycler^®^ 480 software 1.5.0 SP4 for the analysis. Table 1 lists the primers used in this work; all were generated using NCBI Primer-Blast and Primer3 and synthesized from ThermoFisher Scientific and Invitrogen. Each sample was run through real-time PCR three times, and the average CT value was used. The ∆CT value was calculated by comparing the expression of the target mRNA to the mean expression of housekeeping genes (GAPDH and Beta-actin). Each target gene’s relative mRNA expression levels were determined using a 2^−∆∆Ct^ method.

**Table 1:** Real-time quantitative PCR primer sequences.

Genes	Primer sequences	
	FORWARD 5′→3′	REVERSE 5′→3′
Robo4	TTATGGCTCCCTCATCGCTG	GAGGCTGTCTGAGCTGGAAC
ACTA2	AGCCATCTTTCATTGGGATGG	CCCCTGACAGGACGTTGTTA
Fibronectin	GGCCACCATTACTGGTCTGG	GGAAGGGTAACCAGTTGGGG
ICAM-1	CAATTTCTCATGCCGCACAG	AGCTGGAAGATCGAAAGTCCG
S100A4	TTCCTCTCTCTTGGTCTGGTCT	GTCACCCTCTTTGCCTGAGT
SNAI1	GGAGTTGACTACCGACCTTGC	CTGGAAGGTGAACTCCACACAC
VCAM-1	TGAACCCAAACAGAGGCAGAGT	GGTATCCCATCACTTGAGCAGG
Vimentin CD105	AGACCAGAGATGGACAGGTGA CGCAGCCTTACCTCTGGATA	TTGCGCTCCTGAAAAACTGC TCTTTCTGCGAGACTTGTGGG
β -Actin	ATGTGGATCAGCAAGCAGGA	AAGGGTGTAAAACGCAGCTCA
GAPDH	CATGGCCTTCCGTGTTCCTA	GCGGCACGTCAGATCCA

### Protein extraction and western blot analysis

Adherent monolayer ECs were treated with 200 µL of ice-cold RIPA lysis buffer (Beyotime, China), which contained 150 mM NaCl, 50 mM Tris-HCl pH 7.4, 1% NP-40/IGEPAL CA-630, 0.5% sodium deoxycholate, 0.1% SDS, phosphatase and protease (PMSF) inhibitors (Solarbio®, China). Then, adherent cells were carefully scraped and placed in 1.5 ml EP tubes that had previously been sterilized. Protein concentrations were ascertained using a Bicinchoninic Acid (BCA) Protein Assay Kit (Beyotime, China) after the cell lysates were collected at 15,000 rcf for 20 minutes at four degrees Celsius. The protein lysates were then supplemented with Laemmli buffer (Beyotime, China) (one-fourth of the total volume) and heated at 95 °C for 5 minutes. 20 µg of protein lysates were separated using 6–12% SDS-PAGE and blotted in a tank blot machine (Mini-PROTEAN II, Bio-Rad, Hercules, CA, USA) onto polyvinylidene difluoride (PVDF) membranes (Life Science, Germany). Non-fat dry milk (BD, Le Pont de Claix, France) or 5% bovine serum albumin (Solarbio®, China) soaked in a tris-buffered saline solution containing 0.1% (v/v) tween 20 (TBST) (Sigma-Aldrich Co. LLC, St. Louis, USA) was used to block the blotted membranes for 1 hour at room temperature. The membranes were incubated overnight at four degrees Celsius with the primary antibody of interest (Table 2). The membranes were treated with the appropriate horseradish peroxidase (HRP)-conjugated secondary antibodies (CST, Danvers, MA, USA) for 1 hour at room temperature after being washed with 1× TBST. Using the enhanced chemiluminescence (ECL) detection reagent (GE, Healthcare Life Sciences, Little Chalfont, UK), the HRP-immunoreactive bands were detected. Using the Fiji program, the signal intensities of bands were analyzed. β-actin, β-tubulin, or GAPDH protein expressions were used to standardize protein input.

**Table 2:** Antibodies.

Name	Cat. no	Source
IκBα (44D4) Rabbit mAb	#4812	Cell Signaling Technology®
Phospho-IκBα (Ser32) (14D4) Rabbit mAb	#2859	Cell Signaling Technology®
Phospho-PI3 Kinase p85 (Tyr458)/p55 (Tyr199) antibody	#4228	Cell Signaling Technology®
PI3 Kinase p110α antibody	#4255	Cell Signaling Technology®
Akt antibody	#9272	Cell Signaling Technology®
Phospho-SMAD2 (Ser465/Ser467) (E8F3R) Rabbit mAb	#18338	Cell Signaling Technology®
Phospho-Akt (Ser473) (193H12) Rabbit mAb	#4058	Cell Signaling Technology®
β-Tubulin (9F3) Rabbit mAb	#2128	Cell Signaling Technology®
Phospho-NF-κB p65 (Ser536) (93H1) Rabbit mAb	#3033	Cell Signaling Technology®
Phospho-SMAD1 (Ser206) antibody	#9553	Cell Signaling Technology®
Anti-rabbit IgG, HRP-linked antibody	#7074	Cell Signaling Technology®
SLIT2-specific polyclonal antibody	20217-1-AP	Proteintech®
NF-κB p65 polyclonal antibody	10745-1-AP	Proteintech®
GAPDH monoclonal antibody	60004-1-Ig	Proteintech®
Beta-actin polyclonal antibody	20536-1-AP	Proteintech®
Phospho-mTOR (Ser2448) monoclonal antibody	67778-1-Ig	Proteintech®
mTOR monoclonal antibody	66888-1-Ig	Proteintech®
CD31 polyclonal antibody	28083-1-AP	Proteintech®
SMAD2 polyclonal antibody	12570-1-AP	Proteintech®
SMAD1 polyclonal antibody	10429-1-AP	Proteintech®
TGF Beta 1 polyclonal antibody	21898-1-AP	Proteintech®
ROBO4-specific polyclonal antibody	20221-1-AP	Proteintech®
VEGFR2 polyclonal antibody	26415-1-AP	Proteintech®
Collagen type I polyclonal antibody	14695-1-AP	Proteintech®
Fibronectin polyclonal antibody	15613-1-AP	Proteintech®
smooth muscle actin polyclonal antibody	14395-1-AP	Proteintech®
S100A4 polyclonal antibody	16105-1-AP	Proteintech®
Vimentin polyclonal antibody	10366-1-AP	Proteintech®
Recombinant anti-CD105 antibody [EPR21846]	(ab221675)	Abcam
Recombinant anti-VE-Cadherin antibody [EPR18229] - intercellular junction marker	(ab205336)	Abcam
Goat anti-rabbit IgG H&L (Alexa Fluor® 594)	(ab150080)	Abcam
SNAIL antibody	AF6032	Affinity Biosciences
TIE-2 antibody	AF7848	Affinity Biosciences
Claudin-5 antibody	AF5216	Affinity Biosciences
CD31 (PECAM-1) monoclonal antibody (390)	14-0311-82	Thermo Fisher Scientific
ICAM-1/CD54 antibody (G-5)	sc-8439	Santa Cruz
VCAM-1 antibody (1.BB.619)	sc-73252	Santa Cruz

### Immunofluorescence

With or without Robo4 gene manipulation, 20,000 ECs were inoculated on 12 mm round coverslips in 24-well plates and incubated to reach 60–70% confluence at 5% CO_2_ and 37 °C. The ECs were washed in phosphate-buffered saline (PBS) after being exposed to radiation, fixed in 4% paraformaldehyde or 100% ice-cold methanol for 10 minutes at room temperature, and then permeabilized with 0.1% (v/v) Triton X-100 for 15 minutes. After three washes in PBS, the cells were blocked at room temperature for 30 minutes with 1% BSA and 22.52 mg/ml glycine in PBST (PBS + 0.1% Tween 20). At 4 °C, coverslips were incubated overnight with primary antibodies (Table 2). Secondary antibodies conjugated with Alexa Fluor® 594 or Alexa Fluor® 488 (1:500 dilution) (Table 2) were incubated with the cells for 2 hours at room temperature in a damp chamber away from light after the cells had been washed three times with PBST (5 minutes per wash). After thorough rinsing in PBS, the cells were stained with 1 mg/ml of 4′,6-diamidino-2-phenylindoles (DAPI) to label the nuclei. Next, an anti-fade mounting media was used to secure the coverslips, and a Zeiss confocal fluorescence microscope (ZEISS LSM 880, Munich, Germany) was used to detect the fluorescent signals. Pictures of the same antigen were captured with constant acquisition settings to ensure a fair evaluation. We used Fiji software to analyze the captured images (Bethesda, MD, USA). Adobe Illustrator was used to put the figures together.

### Cell counting kit-8 (CCK-8)

96-well plates were seeded with ECs (1 × 10^4^/well) after Robo4 gene editing, and the cells were exposed to 15 Gy gamma radiation. After incubating the culture plate for 4 hours, 10 µL of cell counting kit-8 (Beyotime, Shanghai, China) was applied to the cells 24 hours after IR. Microplate absorbance was read at 450 nm using a Bio Tek Synergy/H1 microplate reader (Winooski, Vermont, USA).

### Wound-healing assay

Cells were maintained in a six-well plate until they reached confluence and wounded with a 200 μL sterile pipette tip perpendicular position to produce a straight scratch. For 48 h, the cell migration distance was determined via an inverted microscope. The images were analyzed by Fiji software, and the cell migration rate was calculated and compared.

### Transwell cell migration assay

Cells (5 × 10^4^) were seeded into the apical chamber (Costar, Cambridge, MA, USA) with 200 µl serum-free DMEM, and DMEM containing 10% fetal bovine serum was added into the basolateral section (Costar, Cambridge, MA, USA). After 24 h, the non-migrated cells were removed by wiping the inward side of the membranes with a cotton swab, and the transmigrated cells that penetrated the membrane were fixed with 4% paraformaldehyde for 20 min and subsequently stained with 0.1% crystal violet for 15 min. The number of transmigrated cells was counted by observation (magnification, ×20) with a Nikon Eclipse Ti inverted microscope, and data were analyzed with Fiji software.

### Capillary tube formation analysis

One hundred microliters of undiluted Matrigel^TM^ (Corning) were layered in a 96-well plate and polymerized for 1 h at 37 °C. Then, ECs were trypsinized, washed with PBS, and added to the precoated wells at 10 × 10^3^ cells per well in a complete medium and immediately irradiated with 15 Gy of γ-radiation. The ECs were incubated for 6 hours at 37 °C in 5% CO_2_ and stained with 2 µg/ml Calcein AM (MK Biotechnology, Shanghai, China). Photographs of at least three fields per well were taken (Nikon NIS Element, ×10 magnification), and total tube length and branching points were quantified using Fiji software.

### Mice

SPF male inbred C57BL/6 mice aged 6–8 weeks were purchased from Beijing Vital River Laboratory Animal Technology Co., Ltd., maintained in specific pathogen-free barrier facilities, and used per protocols approved by the Animal Ethics Committee of Xuzhou Medical University.

### Establishment of a coculture model of ECs and HSCs

Murine microvascular ECs were inoculated in 24-well plates at a density of 2 × 10^4^/ml per well. After adherence for 24 h, ECs were injured with 15 Gy of γ-radiation. The next day, mouse bone marrow cells (BMCs) were collected using Lin-sorting (EasySep™ Mouse Hematopoietic Progenitor Cell Isolation Kit, STEM CELL, Catalog # 19856) and CD117 sorting (CD117 Microbeads, MACS, Cat # 130–091-224). The isolated BMCs were cocultured with ECs according to the optimal culture ratio of 6:1. Primary HSC medium consisted of Stem Span™ SFEM (STEM CELL, catalog number: 09650), SCF (20 ng/ml) (Peprotech, catalog number: 250–03), TPO (20 ng/ml) (Peprotech, catalog number 315–14), and Pen Strep (Gibco, catalog number: 15140–122).

### Cell cycle analysis

Following the coculture of ECs and BMCs, 2 μL each of the corresponding Lin^+^ markers (Ter119, CD45R / B220, CD11b, CD8a, CD4, Gr-1), C-kit and Sca-1 was added to the suspension cells and incubated at 4 °C for 30 min followed by washing. The cells were later fixed and permeabilized with FIX & PERM® Sample Kit A and B (Nordic-MUbio, catalog number: GAS-002 M) for 15 min and subsequently incubated with Ki-67 mouse antibody and DAPI at 4 °C for 30 min and 5 min, respectively. Then, the cell cycle was analyzed using a flow cytometer (BD LSRFortessa; BD Biosciences, San Jose, CA, USA) and Flow Jo software version X.

### Flow cytometry

On Day 7 of coculture, suspending hematopoietic cells were collected and rinsed twice with PBS containing 2% FBS and EDTA (catalog number: R1010, Solarbio, China). To detect lineage negative (LSK) cells, lineage markers (FITC, Ter119, CD45R/B220, CD11b, CD3, CD8a, CD4, Gr-1), Sca-1 (APC, Biolegend, catalog number:108112) and C-kit (PE, BD, catalog number:553355) cocktail was added and incubated with the cells at 4 °C for 30 min in the dark. For lineage-specific markers: Granulocyte: CD11b (FITC, BD, catalog number:557396) and Gr-1 (PE, BD, catalog number: 553126); Megakaryocyte: CD41 (FITC, BD, catalog number:553848) and C-kit (PE, BD, catalog number: 553355); Erythroid: Ter119 (FITC, Biolegend, catalog number: 116206) and CD71 (PE, eBioscience, catalog number:12–0711-82); Lymphocyte: (PE, CD3 and CD45R/B220) were incubated together with the cells. Cell apoptosis was ascertained using an Annexin V staining kit following the manufacturer’s (BD) instructions. The cells were washed and run using a flow cytometer (BD LSRFortessa; BD Biosciences, San Jose, CA, USA), and the data obtained were analyzed by Flow Jo software version X.

### Statistical analysis

Statistics and graphing were performed using GraphPad Prism® 8.0. The analysis findings were shown as the mean ± standard error of the mean (*x* ± SEM). The differences between only two groups were examined through the Mann–Whitney *U* test and analysis of variance (ANOVA) for comparing multiple data sets. Confidence level at *α* = 0.05 (degree of freedom), *P* < 0.05 indicates a statistically significant difference.

## Results

### Robo4 is essential for IR-induced End-MT

**Fig. 1:**
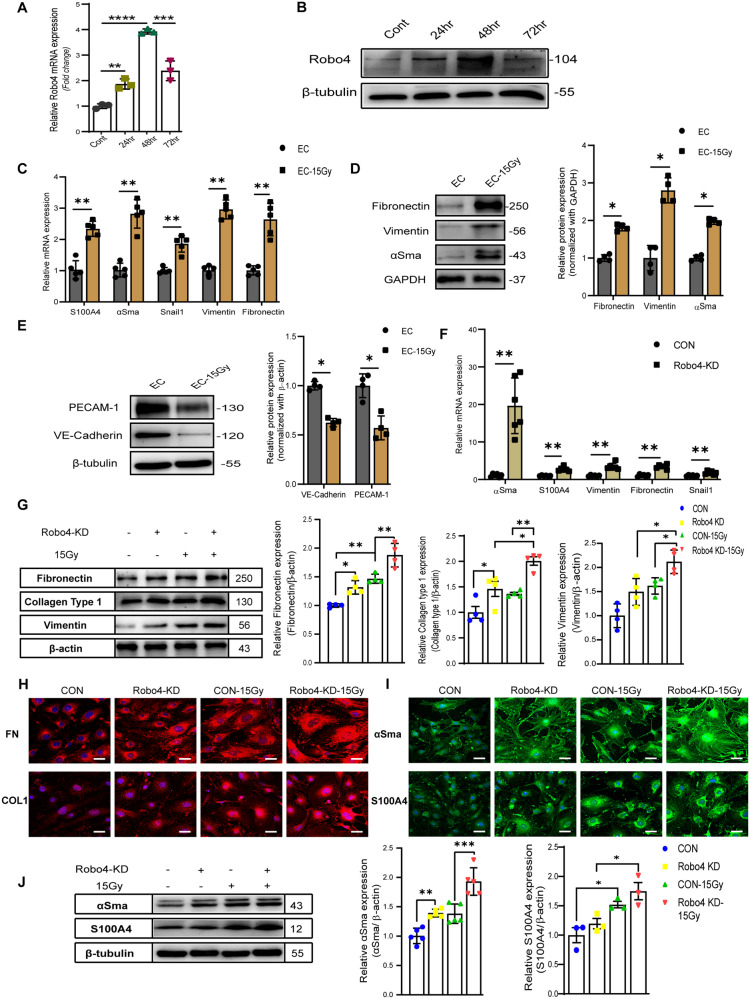
The expression of endothelial and smooth muscle-like cell biomarkers in mouse microvascular ECs is differently regulated after irradiation. ECs were irradiated with 15 Gy of ^137^Cs γ-rays and were harvested at 24, 48, and 72 h post-irradiation. **A**, **B** show representative RT-qPCR and western blot analysis of Robo4 expression levels. Data are mean ± SD; *n* = 3; vs. non-irradiated control. **C** RT-qPCR was used to measure the expression of mesenchymal cell markers after IR treatment. A marked upregulation of αSma, S100A4, SNAI1, Vimentin, and Fibronectin expression was noted following γ-radiation exposure. Data are shown as means ± SEM; *n* = 5. Western blot analysis demonstrated that IR increased Fibronectin, Vimentin, and αSma expression (**D**) and decreased PECAM-1 and VE-Cadherin (**E**). Data are shown as means ± SEM; *n* = 4. After the Robo4 gene knockdown, (**F**) RT-qPCR was used to evaluate the expression of mesenchymal cell markers. Bars indicate the mean fold changes ±SEM relative to the corresponding control; *n* = 6. **G** Western blots and densitometric quantification. **H** Immunofluorescence confocal microscopy of the extracellular matrix components (Fibronectin (FN), Vimentin, and Collagen Type1 (Col1) (red)) protein levels, respectively, in Robo4-depleted ECs. Data are mean ± SEM; *n* ≥ 3. **I** shows immunofluorescence staining and **J** Western blot analysis of αSma and S100A4 (green) in ECs with or without Robo4 knockdown at 48 h post-irradiation. Nuclei stained with 4’,6-diamidino-2-phenylindole (DAPI) appear blue. Values are expressed as mean ± SEM of not less than three independent experiments. Scale bars, 20 µm; *ρ < 0.05; **ρ < 0.01; ***ρ < 0.001; ****ρ < 0.0001).

Upon exposure of murine microvascular ECs (BEND3 cell line) to γ-radiation, we measured the expression of Robo4 1-, 2-, and 3 days post-irradiation. Robo4 expression was upregulated all days after IR, with the highest expression recorded 48 h post-irradiation (Fig. 1A, B). In response to IR, ECs progressively lost the expression of PECAM-1 and VE-Cadherin (Fig. 1E) and gained the expression of mesenchymal markers; fibronectin, vimentin, αSma, and FSP1 (Fig. 1C, D). Also, the Snail1 mRNA level was upregulated (Fig. 1C), suggesting that IR elicits phenotypic and molecular changes reminiscent of End-MT in ECs. We further knocked down Robo4, as confirmed in our previous study [30], and sought the mutual interaction between endogenous Robo4 and End-MT. We found significant upregulation of mesenchymal markers (Fig. 1F). We evaluated End-MT-related proteins by immunoblot and immunocytochemistry analysis and observed increased expression of a myriad of mesenchymal markers, including ECM components (Fig. 1G, H) simultaneously with elevated expressions of αSma and S100A4 (Fig. 1I, J). IR enhanced the levels of End-MT markers in Robo4 silenced ECs (Fig. 1G, J). Interestingly, the End-MT process was accompanied by fibrinogen fiber formation and nuclear translocation of αSma (Fig. 1H, I).

### Transcriptional suppression of Robo4 supports the loss of endothelial identity in response to IR

**Fig. 2:**
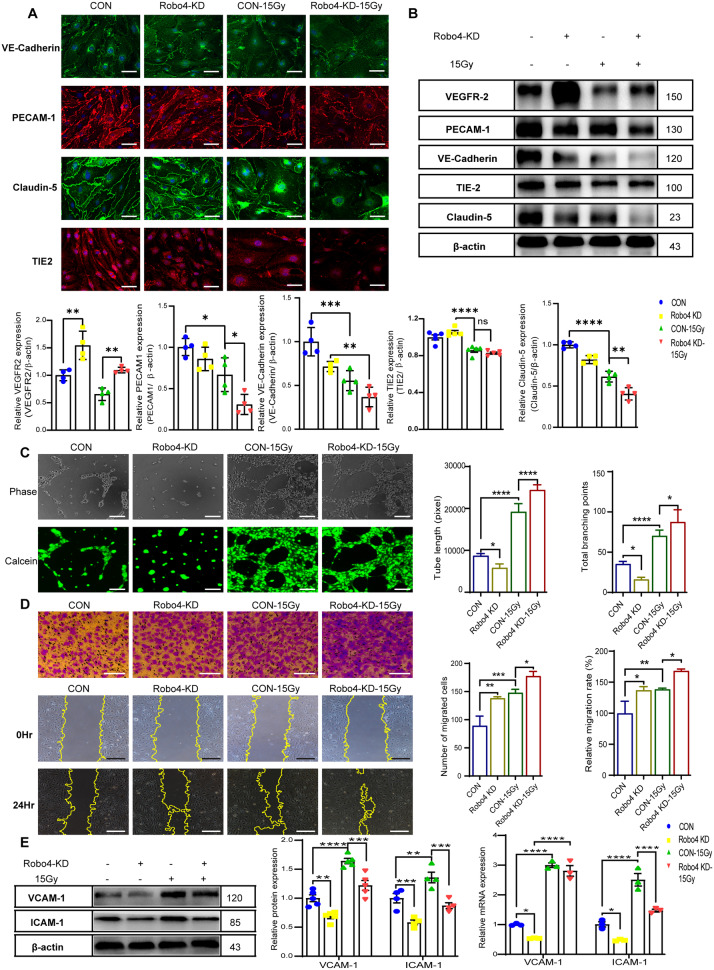
Transcriptional downregulation of Robo4 promotes IR-induced EC migration and pseudo-angiogenesis. **A** Immunostaining of Robo4 silenced murine ECs exposed to γ-radiation in vitro with VE-Cadherin, PECAM-1, Claudin-5, and TIE-2 mouse antibodies; Scale bars, 20 µm. **B** shows a representative blot of endothelial-specific protein levels determined using Western blot and their corresponding densitometric quantifications following Robo4 knockdown and IR. β-actin was used as a loading control; data are shown as mean ± SEM; *n* ≥ 3. **C** Invitro Matrigel-facilitated angiogenesis of ECs after lentiviral-mediated Robo4 downregulation and IR. Bars representing total tube length and branching points are shown as mean ± SEM; *n* ≥ 5. **D** Transwell and wound healing assays measured migration and invasion abilities after Robo4 silencing in ECs with or without IR. The wound closure and migration rate percentage are displayed as the mean ± SEM; *n* = 3. **E** Western blotting and RT-qPCR analysis of endothelial adhesion molecules (VCAM-1 and ICAM-1) in ECs transfected with shRobo4 and the relative control in the presence or absence of IR treatment. Results presented as mean ± SEM. *ρ < 0.05; **ρ < 0.01; ***ρ < 0.001; ****ρ < 0.0001).

Following Robo4 knockdown, we evaluated the expression and organization of endothelial-specific proteins after IR. The data demonstrated apparent alterations in the face of endothelial marker proteins (Fig. 2A, B). The level of Claudin-5 and VE-Cadherin decreased after Robo4 knockdown except for Robo4 antagonist VEGFR2, which was upregulated, and PECAM-1 and TIE-2 exhibited no notable changes in expression (Fig. 2B). 48 h after exposure, IR decreases the expression of endothelial markers. Notably, the combination of Robo4 silencing and IR showed a more significant depletion over mono-treatment. Immunofluorescent staining also confirmed the downregulation of endothelial markers (Fig. 2A). We next performed in-vitro angiogenesis and migration assays and found that Robo4 downregulation reduced the tube formation of ECs. In contrast, IR significantly increased the number of capillary-like structures in Robo4 knockdown ECs (Fig. 2C). Together, these results support a unique role for Robo4 in exerting distinct effects on endothelial capillary formation in response to IR. Our results demonstrate that Robo4 depletion induces End-MT and is considerably enhanced by IR, resulting in an altered phenotype with increased cell migration as shown in wound-healing and Transwell assays (Fig. 2D). ICAM-1 and VCAM-1 mediate the adhesion of cells to the endothelium [33, 34]. Robo4 depletion decreased the expression of ICAM-1 and VCAM-1. We also quantified the expression levels of ICAM-1 and VCAM-1 stimulated by IR (Fig. 2E).

### Enhanced expression of endothelial Robo4 inhibits IR-induced migration and angiogenesis while sustaining the endothelial identity

**Fig. 3:**
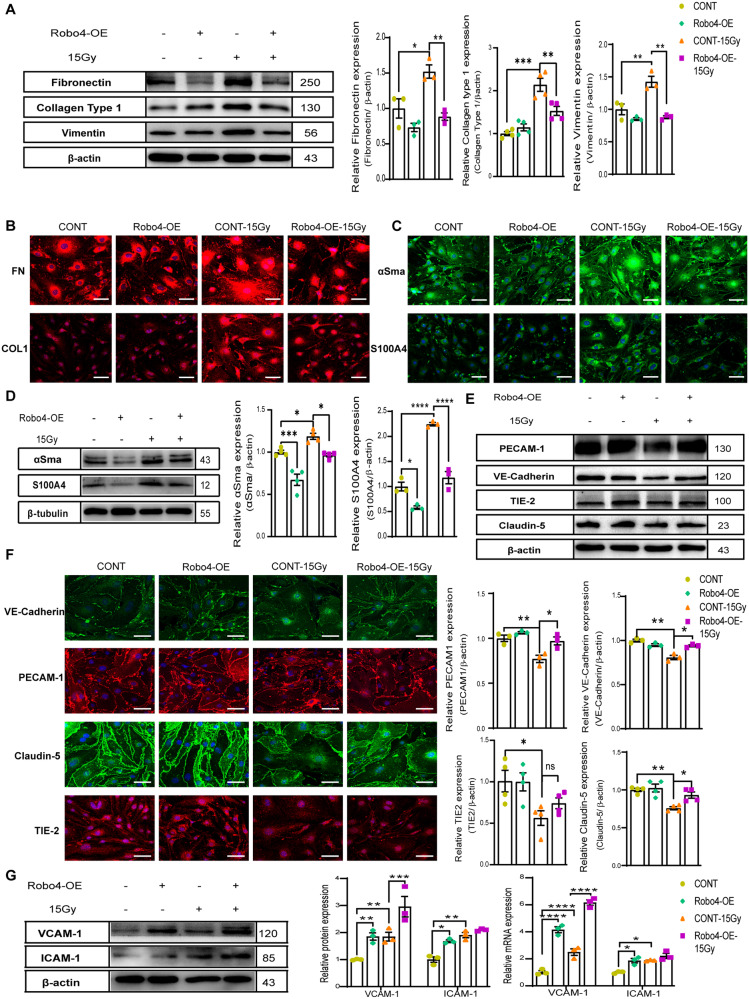
Inhibition of IR-induced Endo-MT upon Robo4 overexpression in ECs. **A** Western blotting for the components of the ECM expression in Robo4 overexpressed murine microvascular ECs, 2 days after radiation exposure to 15 Gy. The bars represent mean ± SEM; *n* = 3. **B** Immunofluorescence staining analysis by Confocal microscopy of ECM components (Fibronectin (FN) and Collagen Type1 (Col1) (red)) and (**C**) other mesenchymal markers (αSma and S100A4 (green)) protein expression in irradiated or unirradiated ECs with or without Robo4 overexpression. Nuclei appear blue (DAPI), and Scale bars represent 20 µm. **D** Relative expression of the smooth muscle-like cell markers: αSma and S100A4 detected by western blotting under 15 Gy dose of γ-radiation following lentiviral-mediated Robo4 upregulation. β-actin was used as an internal control. The graphs represent means ± SEM of at least three independent experiments. **E** Western blot and **F** immunocytochemistry were performed to detect the protein levels of endothelial-specific biomarkers (VE-Cadherin, PECAM-1, TIE-2, and Claudin-5) in mice microvascular ECs treated with or without 15 Gy γ-radiation upon Robo4-enhanced expression. Representative images and quantitative histograms indicated mean ± SEM; *n* ≥ 3. **G** Immunoblotting and qPCR analysis showing VCAM-1 and ICAM-1 expression levels in Robo4 overexpressed microvascular ECs following gamma radiation treatment. Data are mean ± SEM; *n* ≥ 3. Scale bars, 20 µm; *ρ < 0.05; **ρ < 0.01; ***ρ < 0.001; ****ρ < 0.0001).

We aimed to find out if the upregulation of Robo4 could mitigate End-MT induced by IR. After confirming Robo4 overexpression, as reported in our previous study [30], we determined the changes in ECM production. Our data showed no significant differences in the expression of ECM proteins but substantially reduced their levels upon IR (Fig. 3A, B). Next, we sought to determine whether Robo4-enhanced expression affects cytoskeletal structural components associated with mesenchymal cell morphology and motility. WB analysis confirmed reduced expression of αSma and S100A4 even after IR (Fig. 3C, D). Robo4 overexpression did not affect the expression of endothelial markers under control conditions. However, under IR-injury conditions, Robo4 overexpressing cells showed an improved VE-Cadherin, PECAM-1, and Claudin-5 level, except for TIE-2, with no notable changes in expression (Fig. 3E). Immunofluorescence validated these results (Fig. 3F). Additionally, the effect of IR-induced End-MT on endothelial immunoglobulin-like adhesion molecules showed that both VCAM-1 and ICAM-1 expression was upregulated in ECs after Robo4 overexpression and IR.

### Endothelial-specific Robo4 overexpression suppresses IR-induced migration and angiogenesis by regulating the AKT/NF-κB pathway

**Fig. 4:**
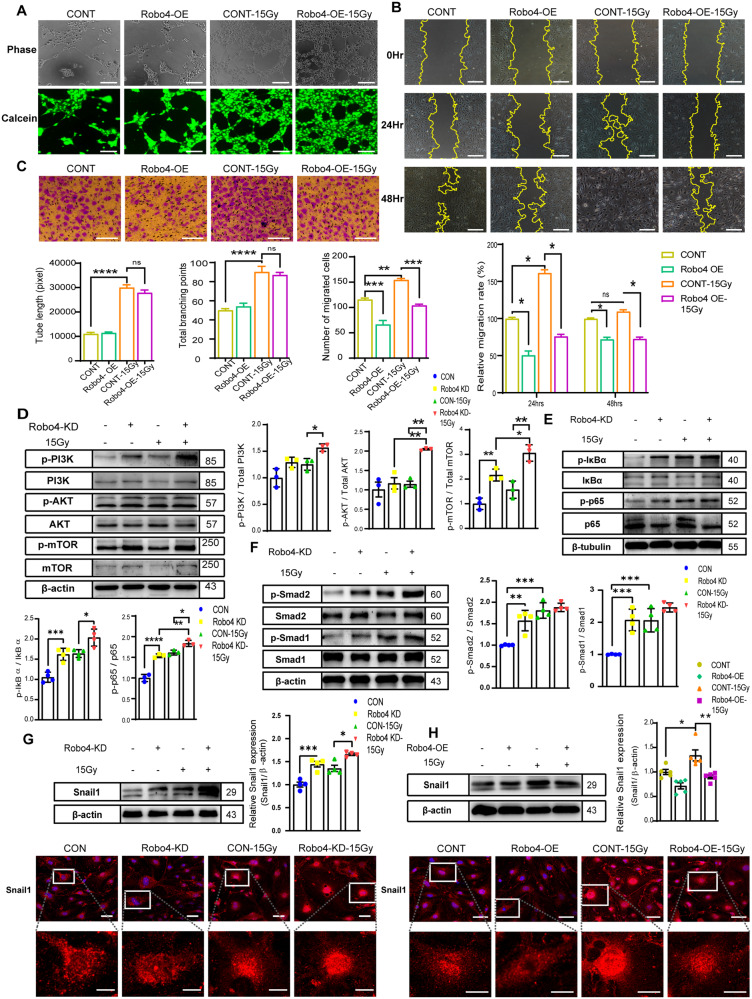
Robo4 modulates Endo-MT through AKT/Smad signaling pathways. **A** Pseudo tube formation assay. The graphs represent means ± SEM of the total tube length and branching point per field determined by quantifying at least 3 areas per well; Scale bars, 200 µm. **B** The migration ability of cells within 48 h was detected by scratch assay. Typical images were obtained at 0 h, 24 h, and 48 h using ×40 amplification, and the migration rate within 48 h was compared as means ± SEM; *n* ≥ 3. **C** Transwell assay indicates that Robo4 overexpression inhibits IR-induced migration of ECs. The analytical data are presented as mean ± SEM; *n* ≥ 3. **D** After the cells were transfected with Lentiviral shRobo4 and shControl in the presence or absence of ionizing radiation, the proteins in the PI3K/AKT/mTOR pathway of the ECs were evaluated using western blot. Data are the mean of three independent experiments, *n* ≥ 3. Error bars represent SEM, and data were analyzed by one-way ANOVA and Tukey’s post hoc test. **E** Immunoblots and quantification of phosphorylated p65 (NF-κB) and IκBα expressions in ECs with or without Robo4 depletion and IR. Data are shown as means ± SEM; *n* ≥ 3. **F** Protein expression levels of p-Smad1, Smad1, p-Smad2, and Smad2 in ECs treated with shRNA for Robo4 and ionizing radiation. Data are the mean of three independent experiments, and error bars represent SEM. Expression of Snail1 was detected by immunoblotting and Immunofluorescence staining in microvascular ECs lacking (**G**) or (**H**) overexpressing Robo4 treated with γ-radiation. Data are shown as means ± SEM; *n* ≥ 3. Scale bar, 20 µm; *ρ < 0.05; **ρ < 0.01; ***ρ < 0.001; ****ρ < 0.0001).

We assessed the degree of angiogenesis and migration of ECs after Robo4 overexpression under IR-induced stress conditions. ECs did not exhibit notable changes in angiogenic potential upon enhanced Robo4 expression. Still, potently inhibited tube formation stimulated by γ-radiation (Fig. 4A). Migration assays revealed that Robo4 overexpression significantly reduces EC motility induced by γ-radiation (Fig. 4B, C). We sought to identify the signaling pathway mediating this effect. We next explored the indispensability of PI3K/AKT/mTOR signaling in the modulation of EC integrity following Robo4 gene silencing and γ-radiation. We examined the expression of t-PI3K/p-PI3K, t-Akt/p-Akt, and t-mTOR/p-mTOR. The results showed that, after Robo4 knockdown, the p-PI3K, p-AKT, and p-mTOR levels increased (Fig. 4D). As expected, our results confirmed that Robo4 downregulation highly promotes IR-induced activation of the PI3K/AKT/mTOR pathway in ECs. We further examined the activation of NF-κB-p65 and its inhibitor, IκBα. The results showed that shRobo4 led to the phosphorylation of IκBα and activation of NF-κB-p65. In addition, shRobo4 and IR significantly stimulate the activation of p65, which may lead to changes observed in endothelial functions (Fig. 4E). These results suggest that Robo4 depletion promotes IR-induced End-MT partly by upregulating the AKT/NF-κB pathway.

### Silencing Robo4 in murine microvascular ECs activates Smad1/2 pathways and promotes nuclear translocation of Snail1

We further explored the influence of Robo4 on the proteins related to the canonical Smad signaling, and our data showed that Robo4 depletion induced apparent phosphorylation of Smad1/2, which is elevated by IR (Fig. 4F). We next analyzed Snail’s total cellular expression levels and nuclear translocation. Robo4 knockdown can highly potentiate Snail1 expression and nuclear translocation induced by IR (Fig. 4G). We further investigated whether Robo4 could modulate the Snail1 to mitigate IR-induced End-MT. Our results showed that Robo4 overexpression exhibited no significant change in Snail production but could reduce the expression and nuclear translocation of Snail caused by IR (Fig. 4H).

### Robo4 interacts with endoglin to inhibit IR-induced activation of Smad/AKT signaling pathways of Endo-MT

**Fig. 5:**
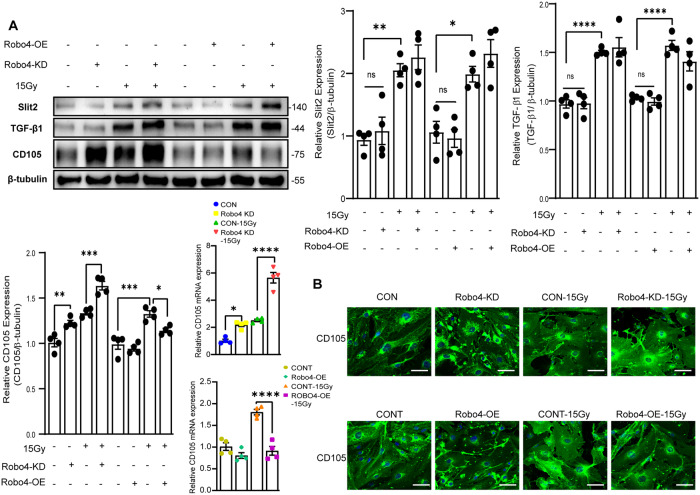
The synergetic effect of Robo4 and Endoglin through AKT/Smad pathways regulates IR-induced Endo-MT. **A** Western blot analysis of Slit2, TGF-β1, and CD105(endoglin) in response to Robo4 gene manipulation and IR using Slit2, TGF-β1, and CD105 antibodies in Robo4-KD and Robo4-OE endothelial ECs under monolayer culture conditions. Data are representative of at least three independent experiments. Densitometric analysis was based on CD105, Slit2, and TGF-β1 band intensities, normalized internally with β-actin. Next, the representative plots have assessed the mRNA expression of CD105 following modification of the Robo4 gene and exposure to irradiation. **B** Immunofluorescence staining with anti-CD105 in ECs. Representative photographs are shown; Scale bar, 20 µm. *ρ < 0.05; **ρ < 0.01; ***ρ < 0.001; ****ρ < 0.0001).

We next sought to determine the function of Robo4 in regulating TGF-β family proteins upstream of the canonical and non-canonical pathways of End-MT. Our results indicated that IR-induced TGF-β and Slit2 expression. Conversely, Robo4 gene manipulation does not significantly alter TGF-β and Slit2 levels (Fig. 5A). Next, we checked if coreceptors of TGF-β family proteins could be involved in the Robo4-mediated modulation of End-MT. Our data revealed that Robo4 knockdown inversely correlates with endoglin (CD105) expression. Simultaneously, Robo4 deficiency promotes the elevation of endoglin protein levels following exposure to γ-radiation. Of note, Robo4 overexpression reduces endoglin expression and inhibits its upregulation induced by IR (Fig. 5A, B). These results suggest that endoglin may interact with Robo4 to regulate IR-induced End-MT.

### EC Robo4 knockdown in the IR-injured endothelium inhibits the expansion of HSPC and promotes HSC quiescence and apoptosis

**Fig. 6:**
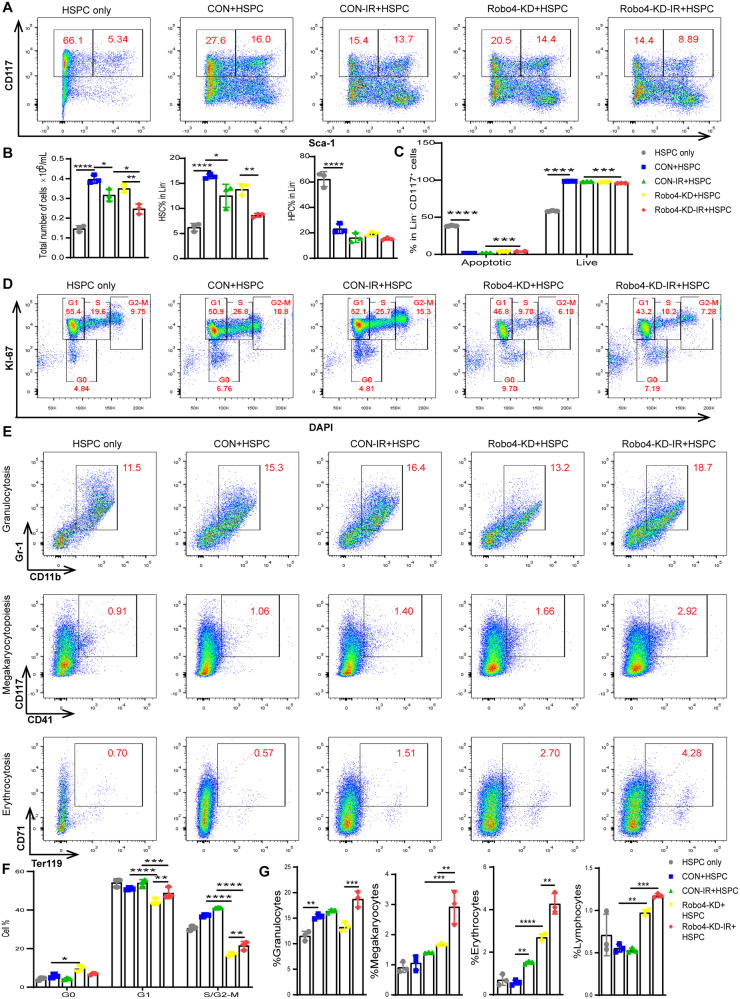
Analysis of injured Robo4 knockdown ECs on HSC expansion in a coculture system. **A**, **B** Flow cytometry analysis of HSC frequency after coculture with or without Robo4 knockdown and IR-injured ECs. **C** Flow cytometric analysis of apoptosis induced by injured endothelial monolayer on HSC using Annexin V and 7-AAD. **D**, **F** HSC cell cycle analysis by flow cytometry. The effect of endothelial Robo4 silencing on HSC differentiation and maturation after IR injury was also determined. After seven days, results were collected after HSC coculture with normal or injured Robo4-depleted ECs. **E**, **G** Flow cytometry analysis of the proportion of granulocytes, megakaryocytes, erythrocytes, and lymphocytes (All data are analyzed as means ± SEM; *n* = 3, *ρ < 0.05; **ρ < 0.01; ***ρ < 0.001; ****ρ < 0.0001).

We tested the hypothesis that Robo4 depletion in ECs impairs hematopoietic regeneration. We cocultured murine HSPC with invitro expanded murine microvascular ECs in which the Robo4 gene transcription was suppressed. Flow cytometric analysis validated the function of ECs in support of hematopoietic reconstruction in the mimicked BM microenvironment. However, IR-injured ECs showed a reduced ability to sustain HSPC typical functionalities ex vivo adequately. HSPC cultured on Robo4-deficient ECs demonstrated that Robo4 knockdown promotes quiescence of HSC regardless of IR (Fig. 6A, B). The presence of ECs inhibits HSPC apoptosis ex vivo. However, injured Robo4-deficient ECs promote apoptosis of HSPC (Fig. 6C). The frequency of LSK cocultured on Robo4-depleted ECs was significantly higher than its control. We investigated the fraction of LSK in G0. As expected, the frequency of LSK quiescent cells was higher when cultured with Robo4-deficient ECs. Conversely, we also found that the frequency of LSK in the cell cycle’s proliferative phase (S/G2/M) is significantly lower, with ECs lacking Robo4 (Fig. 6D, F). Functionally, we detected the proportion of granulocytes, megakaryocytes, erythroid cells, and lymphocytes. HSPC cultured on Robo4 silenced ECs demonstrated myeloid and lymphoid differentiation. The differentiation was further exacerbated when the ECs were irradiated before the coculture with HSPC (Fig. 6E, G).

### EC Robo4 overexpression inhibits the effect of injured endothelium on HSC and promotes their expansion

**Fig. 7:**
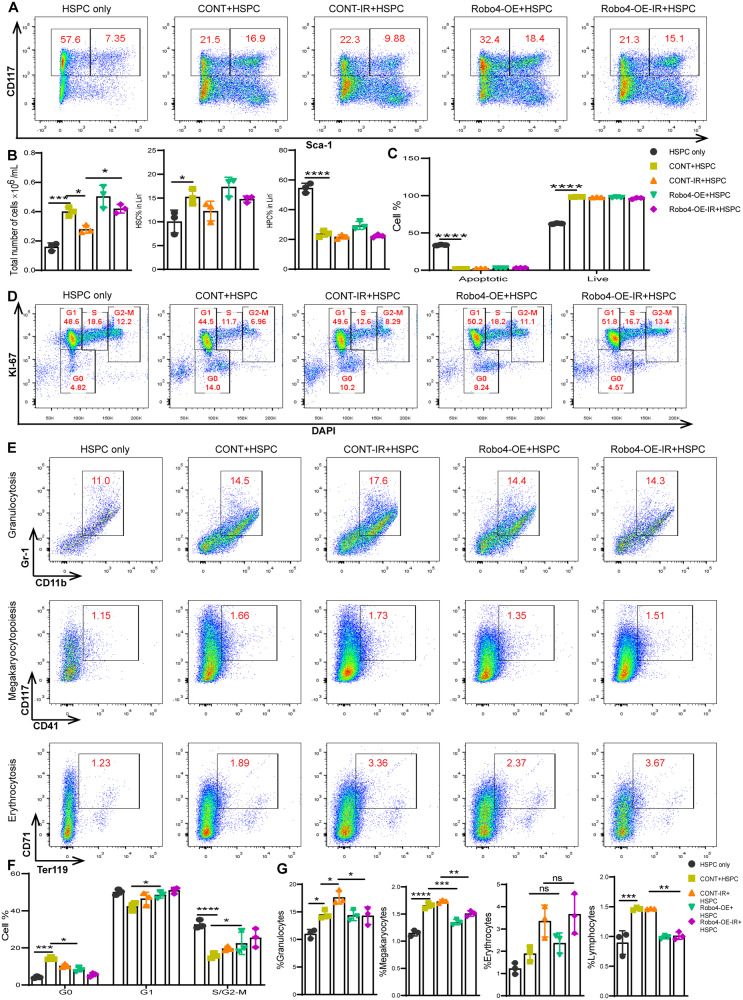
Evaluation of endothelial Robo4 overexpression on HSC function in a coculture system after IR. **A**, **B** representative flow cytometric analysis of HSC proportion after coculture with normal or injured Robo4 overexpressing microvascular ECs. **C** Flow cytometric analysis of apoptosis induced by injured endothelial monolayer on HSC using Annexin V and 7-AAD. **D**, **F** HSC cell cycle analysis by flow cytometry using Ki-67 mouse antibody and DAPI. Examining the impact of endothelial Robo4 overexpression on HSC maturation and differentiation during injury induction. After seven days, results were obtained after HSC coculture with normal or injured Robo4 overexpressing ECs. **E**, **G** Flow cytometry analysis of the proportion of granulocytes, megakaryocytes, and erythrocytes (Data are analyzed and shown as means ± SEM; *n* = 3, *ρ < 0.05; **ρ < 0.01; ***ρ < 0.001; ****ρ < 0.0001).

HSPC cultured on Robo4-enhanced ECs resulted in an absolute increment in nucleated cells. However, the proportion of LSK showed that Robo4 overexpression in ECs mitigates the effect of IR-induced injury on HSC and promotes the expansion of HSC (Fig. 7A, B), suggesting that Robo4 in BM ECs may protect HSC from radiation injury effects. At the same time, there is no change in the apoptotic states of HSPC (Fig. 7C). We also investigated the fraction of HSPC in the quiescent and proliferative phases of cell cycles. We observed a reduced frequency of HSPC quiescent cells cultured on Robo4-OE ECs. Moreover, we found that Robo4 overexpression in ECs supports HSPC expansion, as the frequency of HSPC in the proliferative stage of cell division was substantially higher even in the presence of injured ECs (Fig. 7D, F). Also, Robo4 upregulation in ECs does not considerably affect HSC differentiation ex vivo except in megakaryocytes and lymphoid cells. Interestingly, irradiated Robo4-enhanced ECs less affected HSC differentiation and maturation (Fig. 7E, G). These results suggest that EC Robo4 could efficiently control HSC differentiation ex vivo.

## Discussion

In response to IR, BM EC gene expressions undergo rapid alterations resulting in defects, including vessel dilation and leakiness in the hematopoietic-supportive BM [35, 36]. There is room for improvement in current HSCT outcomes and radiotherapy-induced myelosuppression if we can better understand the processes that protect the BM endothelium after injury. Growing evidence points to the developmental End-MT process directly involved in vascular injuries [37, 38]. Our data confirmed that IR compromises the integrity of the microvessels by inducing End-MT. Vascular endothelium subjected to IR and chemotherapy is characterized by cell death and the acquisition of a long-lasting activated phenotype responsible for vascular dysfunction [39]. However, the involvement of IR in End-MT is not utterly new, as it has been demonstrated in irradiated human aortic, pulmonary, intestinal, and umbilical cord ECs [40, 41].

Along with increased expressions of specific mesenchymal cell markers after IR exposure, we also observed Robo4 upregulation. Therein, we proposed that Robo4 may be involved in the modulation of vascular integrity by regulating End-MT induced by IR. However, no research has been conducted on Robo4’s role in modulating End-MT following IR.

This study showed that Robo4 deficiency potentiates murine microvascular ECs toward End-MT. End-MT is observed with increased ECM production and fibrosis in various chronic illnesses [42]. The loss of endothelial phenotypes, including spindle morphology and intercellular adhesion, and the acquisition of mesenchymal characteristics, such as increased rate of migration and angiogenesis, are the main changes associated with End-MT [10]. These cells produce ECM, which contributes to structural remodeling. Huang et al. demonstrated that choroid-retinal ECs migrated less when Robo4 was silenced [43]. Contrarily, others also reported that the ability of ECs to migrate and form tubes was enhanced when Robo4 expression was inhibited in HBMECs [44]. Both loss of function and overexpression of Robo4 in HUVECs dramatically slowed wound healing and led to irregular tube networks in vitro [45]. As a result, Robo4’s functionality depends on the environment and the type of cell or tissue involved.

Previous research has shown that PI3K and Akt inhibition can prevent αSma expression [46]. Others have demonstrated that IR promotes in vitro angiogenesis by facilitating the phosphorylation of PI3K, AKT, and mTOR in ECs [47]. Also, by blocking the VEGR2-mediated PI3K/AKT pathway, Robo4 reduces EC migration and angiogenesis [44]. Our study provided evidence suggesting that silencing Robo4 would activate the PI3K/Akt/mTOR pathway, accelerating IR-induced End-MT.

A previous study has demonstrated that TNF-α stimulates End-MT in the endothelium by activating Akt/NF-κB [48]. Additionally, hyperglycemia induces End-MT through the Akt/PI3K/NF-kB pathway and is linked to fibrosis in diabetic patients [49]. Other studies have also demonstrated that the inhibition of PI3K and AKT effectively prevents TGFβ or arsenic-trioxide-induced End-MT [50, 51]. IR induces the release of pro-inflammatory cytokines such as TNF-α, IL-1β, and IL-6, which may then activate TGF-β driven End-MT via the NF-kβ pathway [52–54]. Robo4 suppression increased p65 phosphorylation in this work, which is imperative for its nuclear translocation. Others have shown that TNF-α induces Robo4 expression via an interaction between the NF-κB heterodimer, p65-p50, and the NF-κB motif in the Robo4 upstream promoter [55]. The NF-κB family is an essential class of transcriptional regulators that mediate the functions of ECs in a regular or inflammatory state. Furthermore, AKT participates in NF-κB activation by mediating IKKA phosphorylation, activating its downstream target IκB [11, 54]. In the current study, Robo4 depletion increased AKT, IκB, and p65 phosphorylation [56]. These findings suggest that Robo4 knockdown promotes End-MT by positively regulating the AKT/NF-κB pathway. Moreover, AKT can interact with unphosphorylated Smad3 directly and inhibit IR-induced EMT [57]. Studies have demonstrated that NF-κB binds to the promoters of End-MT-related genes like Snail, Slug, and Twist, increasing their transcription [10, 11]. The current study also discovered that the crosstalk between Smad and Akt/NF-κB signaling might be essential in this transition process.

Previous research has indicated that Smad pathway inhibitors that act downstream of TGF-β may be needed to reverse End-MT and, by extension, inhibit BM fibrosis [58]. In endotoxemia, vascular permeability is reduced when Robo4 expression is upregulated by Smad signaling [59]. However, other reports have demonstrated that Smad2/3 silencing blocked TGF-β2’s effect on endothelial dysfunction [60]. This study discovered insufficient Robo4 expression promotes Smad1/2 phosphorylation in ECs after IR. The suppression of angiogenesis following Robo4 knockdown in vitro may be attributable to the fact that TGF-β-ALK5-Smad2 signaling has been shown to keep the endothelium quiescent, thereby inhibiting angiogenesis [61].

We found no altered TGF-β1 responses upon Robo4 gene manipulation, indicating that Robo4 may interact with other proteins upstream of the putative pathways to regulate End-MT. Endoglin is a coreceptor for TGF-β, and its expression is upregulated in proliferating and injured ECs [62]. Our data show that Robo4 suppresses endoglin expression, which may inhibit IR-induced Smad1/2 phosphorylation. Comparatively, several studies present contradictory information on the function of endoglin in controlling TGF-β-mediated Smad1/5 and Smad2/3 signaling in various cell types. For instance, one study found that endoglin ectopic expression in rat myofibroblasts increased Smad2 phosphorylation [63], whereas other studies found no effect on TGF-β-induced phosphorylation of Smad1 or Smad2 [64–66]. In human chondrocytes, it has been reported that endoglin reduces TGF-β1/ALK5-induced Smad3-driven transcriptional activity and ECM formation [67]. Endoglin functions as a positive regulator of both ALK1-induced Smad1 and ALK5-induced Smad2 activation in BM stromal cells [68]. Also, endoglin interacts with the PI3K to activate Akt signaling in ECs [69]. Our current findings indicate that Robo4 interacts with endoglin to regulate IR-induced Smad1/2 and AKT activation of End-MT.

Depending on the cell environment, TGF-β promotes Snail activation through both Smad-dependent and Smad-independent pathways [20]. Evidence shows that inhibiting Robo4 in human aortic ECs increases αSma and Snail1 expression [70]. Our results suggest that Robo4 via Smad or AKT pathways promotes Snail1 activation and nuclear translocation to regulate IR-induced End-MT.

Like our previous study on another anti-angiogenic molecule, PEDF [71], Robo4 in ECs ameliorates the IR-induced inhibitory effect on HSC functions in our coculture model. HSC has various biological characteristics, the most important of which is the ability to self-renew and infinitely proliferate [72]. Our data revealed that treatment of ECs with γ-radiation can inhibit the HSC cell cycle. This study also investigated the effect of IR-induced endothelial injury on HSC differentiation. Our previous work has demonstrated that busulfan-injured ECs can promote the differentiation and maturation of HSCs [71]. Consistently, our present results indicate that IR-induced EC injury inhibits self-renewal and supports the multi-lineage differentiation potential of HSCs.

IR is associated with increased hypoxia and elevated reactive oxygen species (ROS) levels within the BM. The mitochondrial damage caused by IR contributes to increased intracellular ROS levels and hypoxia [7]. When exposed to hypoxia, the Robo4 gene was overexpressed in ECs [73]. Excessive ROS can activate inflammatory signaling, perpetuating HSC dysfunction. For example, elevated levels of ROS have been shown to impair HSC engraftment potential and promote myeloid-biased differentiation via activation of the mTOR [74]. Also, another report suggests that suppressing NF-κB-dependent vascular inflammation radio-protects the BM microenvironment [75, 76]. Interestingly, our findings indicate that silencing endothelial Robo4 favors IR-induced differentiation of HSCs.

Increased surface expression of adhesion molecules like VCAM-1 and ICAM-1 is a hallmark of activated ECs [77]. Previous research has shown that deleting Robo4 from the germline causes defective HSC trafficking by downregulating VCAM-1 protein on sinusoidal ECs [78]. Another study revealed that ICAM-1-deficient mice exhibited a significant expansion of long-term HSCs with impaired quiescence and a preference for the proliferation of myeloid cells [79]. These works imply that increased adhesion molecules on ECs may facilitate the recruitment and homing of HSPCs to the BM after myeloablative preconditioning and HSPC transplantation. Robo4 overexpression in ECs in our coculture system upregulates the expression of adhesion molecules, thereby preserving HSC stemness and normal functions following IR preconditioning. As we look for mechanisms underlying hematological recovery after myelosuppressive injury, Robo4’s role in modifying IR-induced End-MT makes it the perfect candidate for further investigation.

**Fig. 8:**
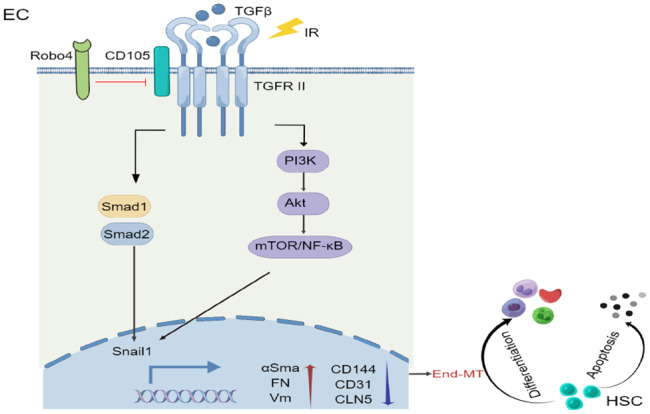
Schematic illustration of Robo4 inhibiting irradiation-induced End-MT through the regulation of CD105/Smad and CD105-AKT/NF-κB signaling pathways. In the physiological situation, ECs are stable; however, this stability is perturbed in a pathological state. Irradiation injury can activate the canonical (TGF-β1/Smad) and non-canonical (TGF-β1/AKT/ NF-κB) pathways, which promote Snail1-mediated End-MT. Robo4 downregulates endothelial CD105 (endoglin) expression (a coreceptor for TGF-β family proteins) to modulate irradiation-induced End-MT, which supports HSC apoptosis and differentiation.

In conclusion, the findings of this work revealed that Robo4, via the endoglin-Smad and endoglin-AKT signaling pathways, is a critical regulator of End-MT, a process that, in the presence of pathological or physiological stressors, affects the integrity of the vasculature and, thus, HSC activities in the BM microenvironment (Fig. 8).

### Supplementary information

WB raw data

## Data Availability

All data generated or analyzed during this study are included in the paper.
