# Supplementary material for: Endothelial Robo4 suppresses endothelial-to-mesenchymal transition induced by irradiation and improves hematopoietic reconstitution
Source: Cell Death Dis. 2024 Feb 21;15(2):159. doi: 10.1038/s41419-024-06546-4 (PMC10881562; doi:10.1038/s41419-024-06546-4)

Figure 1. Western Blot Raw Images

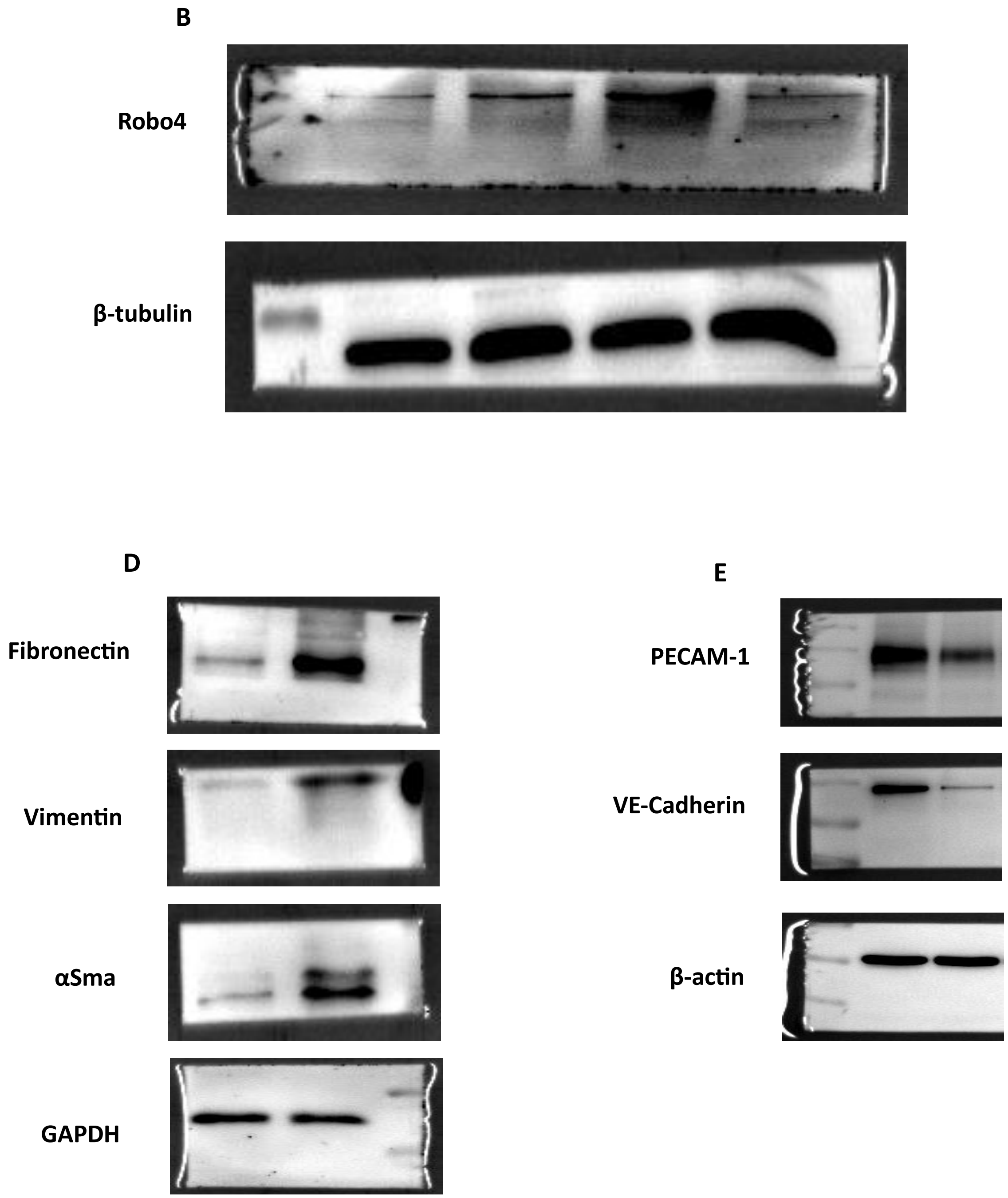

Figure 1. Western Blot Raw Images

G

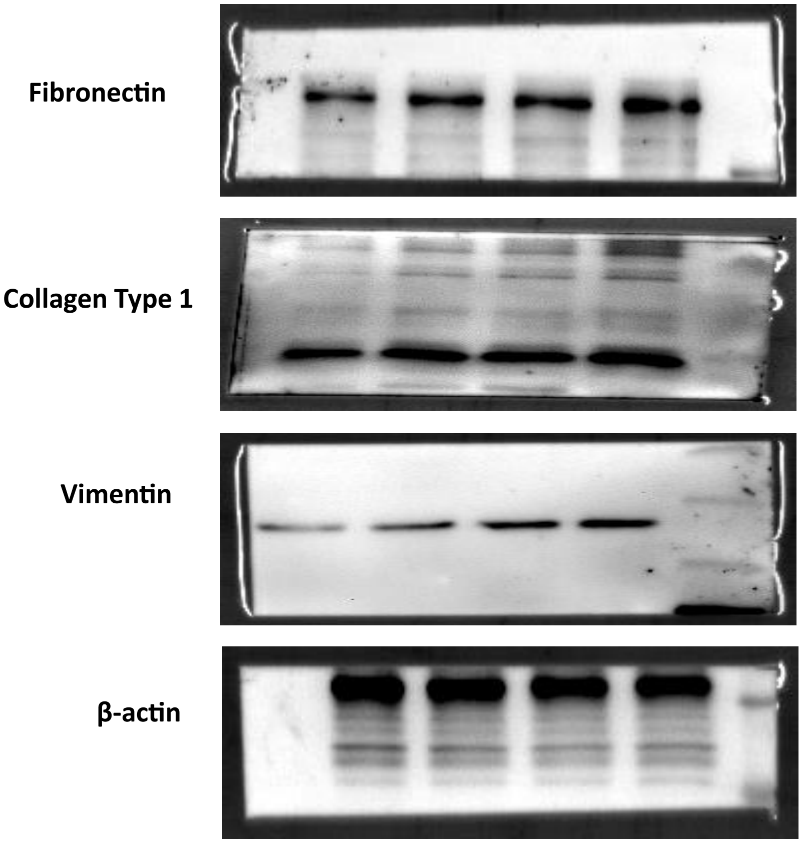

J

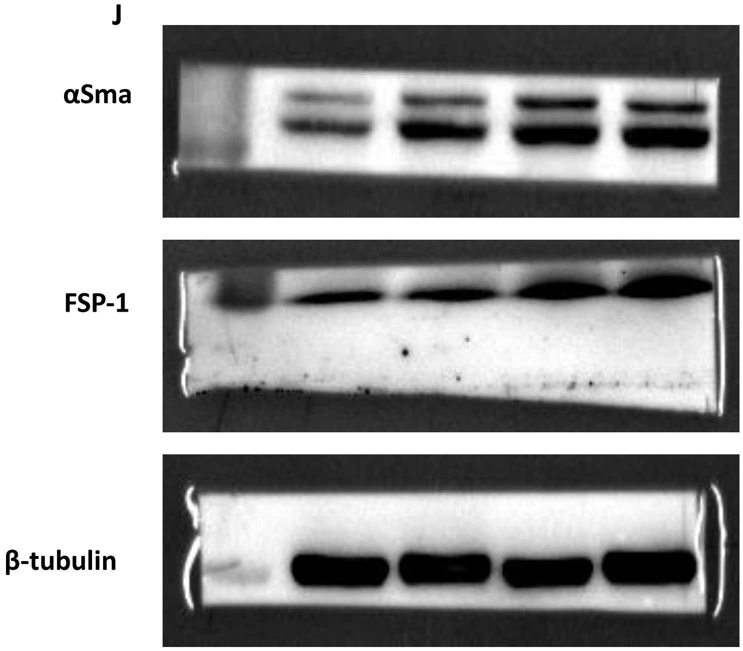

Figure 2. Western Blot Raw Images

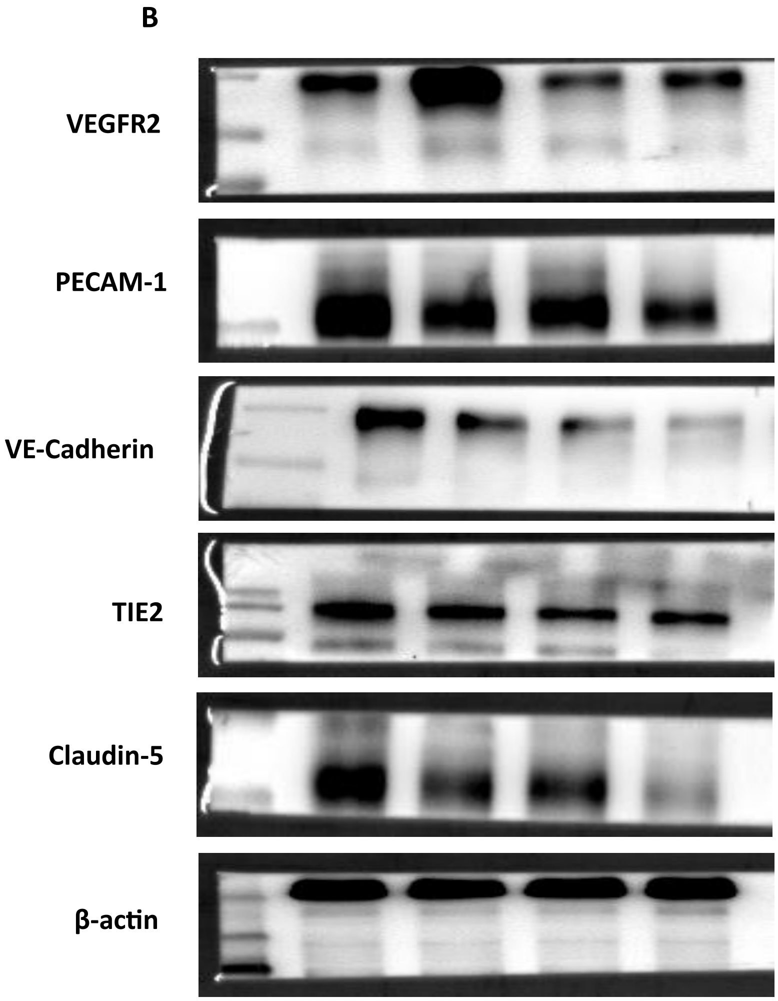

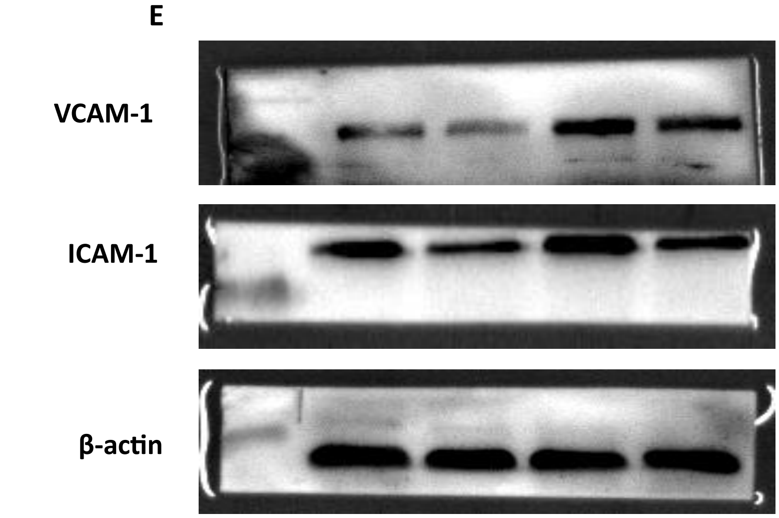

Figure 3. Western Blot Raw Images

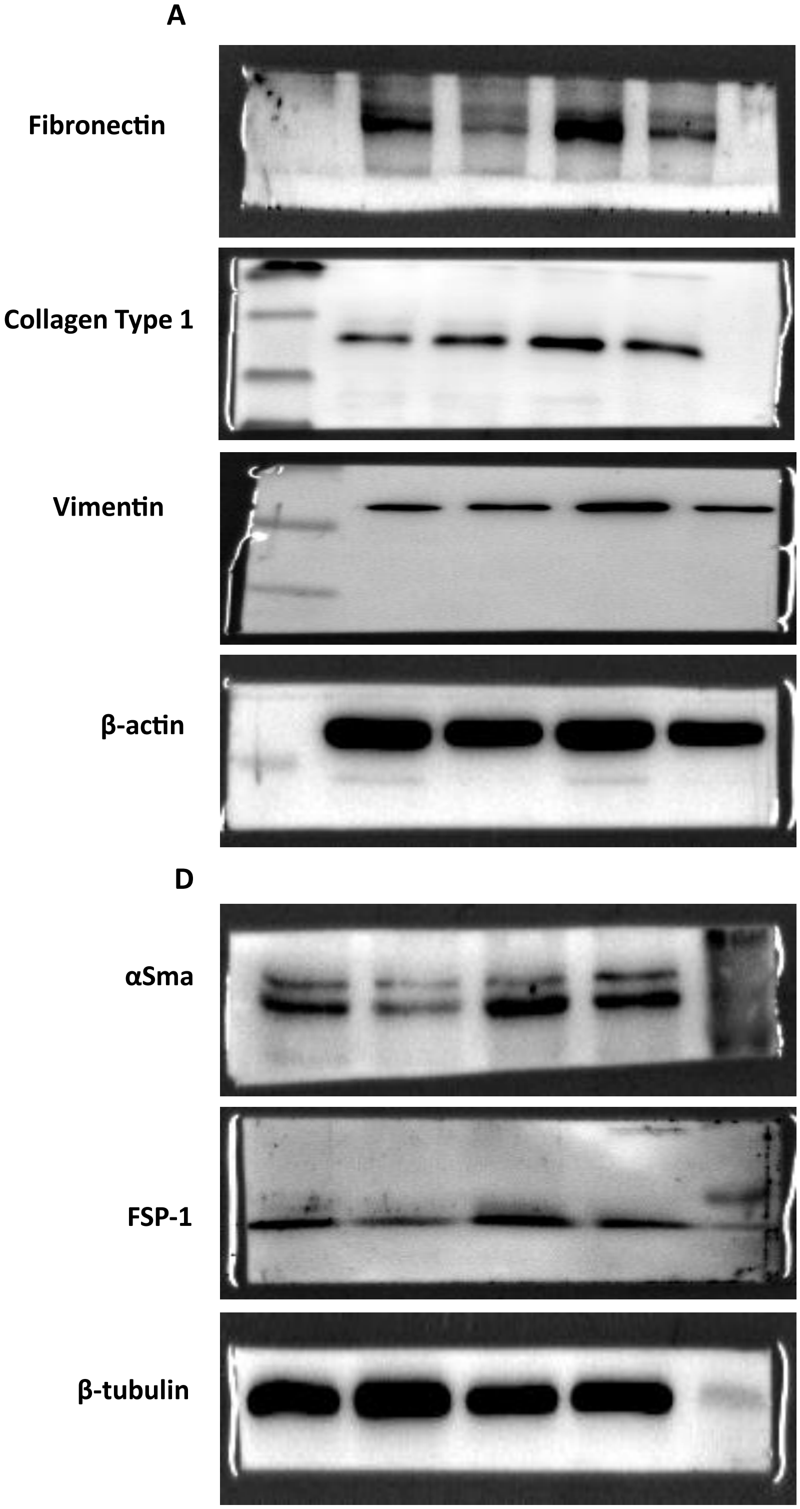

Figure 3. Western Blot Raw Images

E

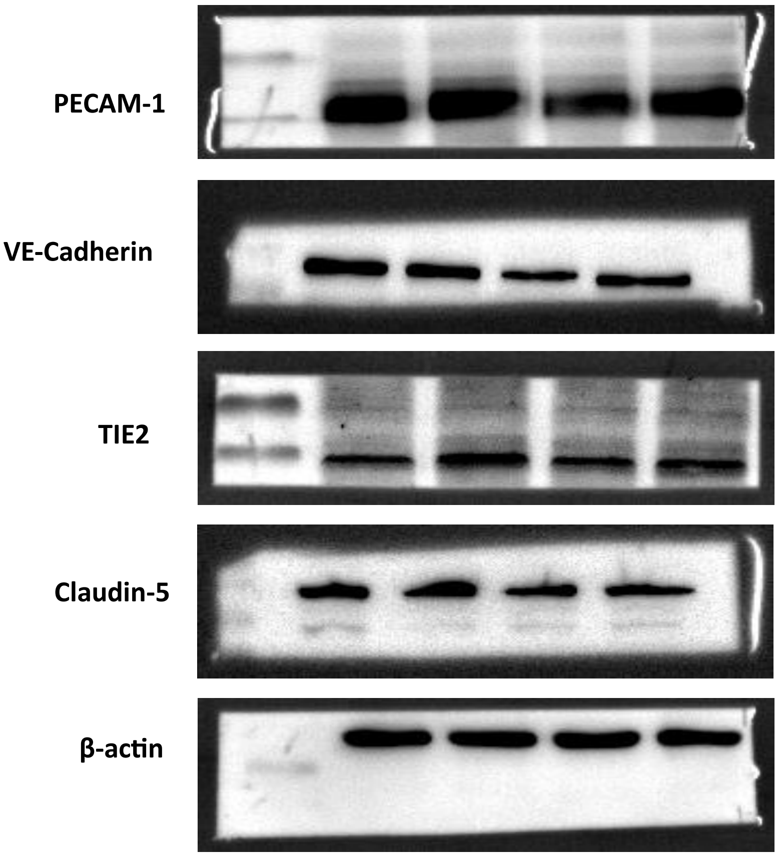

G

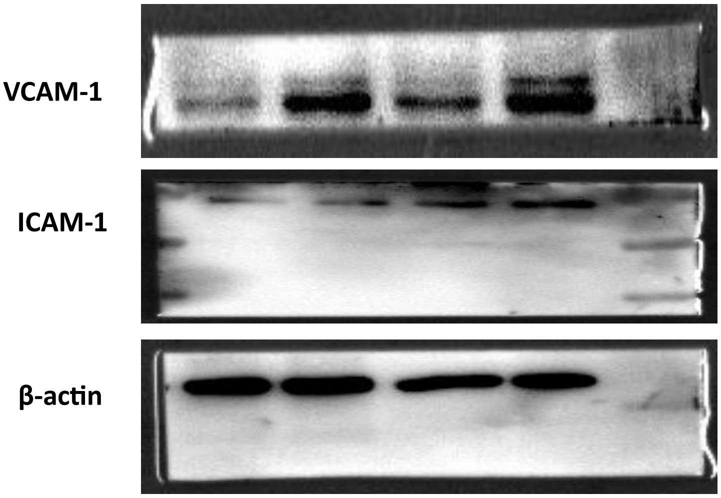

Figure 4. Western Blot Raw Images

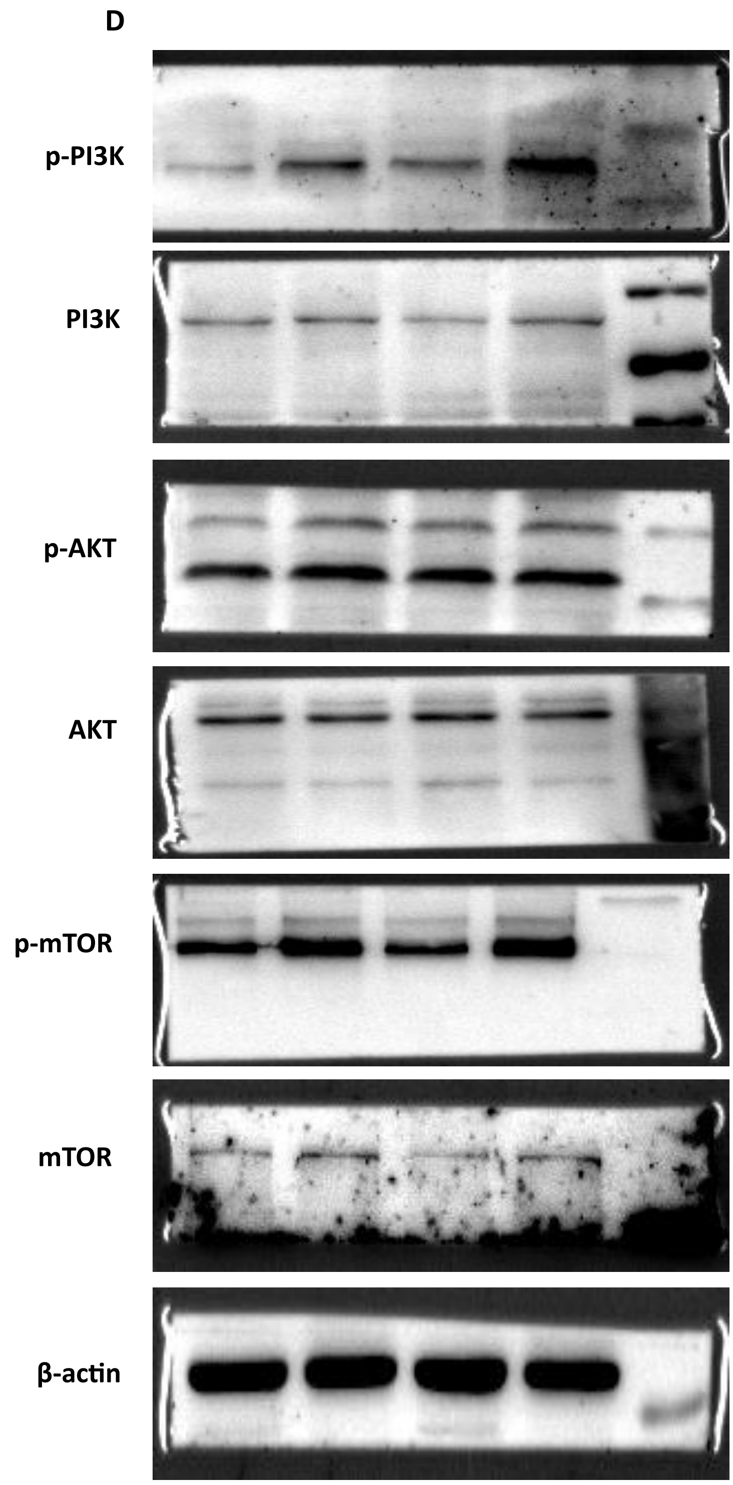

Figure 4. Western Blot Raw Images

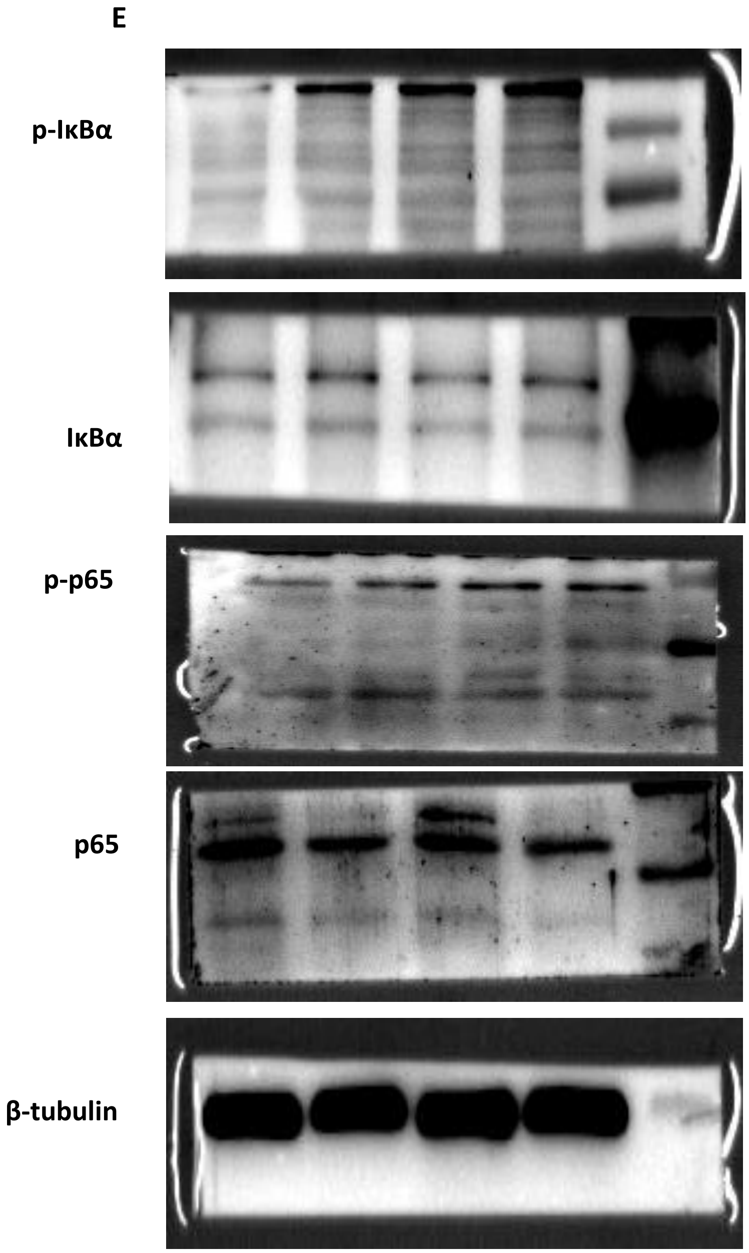

Figure 4. Western Blot Raw Images

F

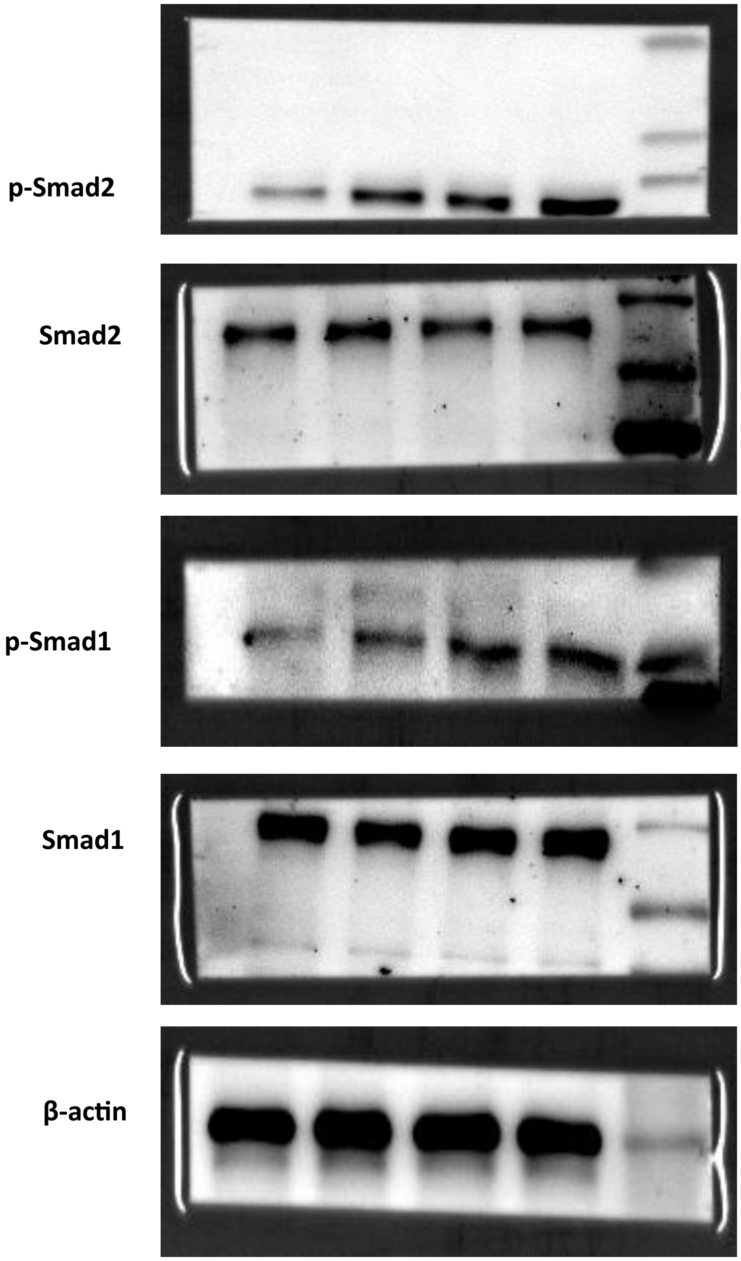

Figure 4. Western Blot Raw Images

G

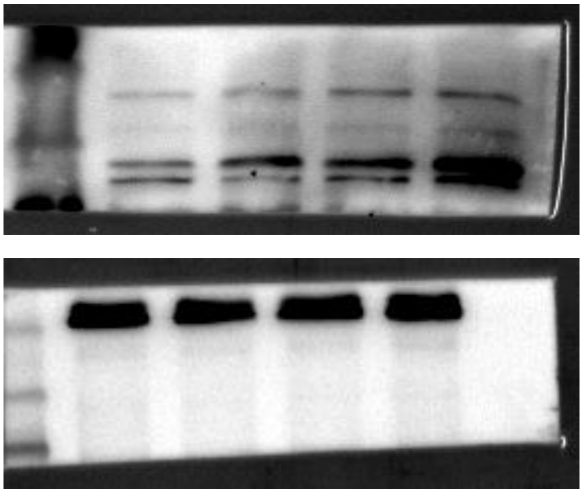

H

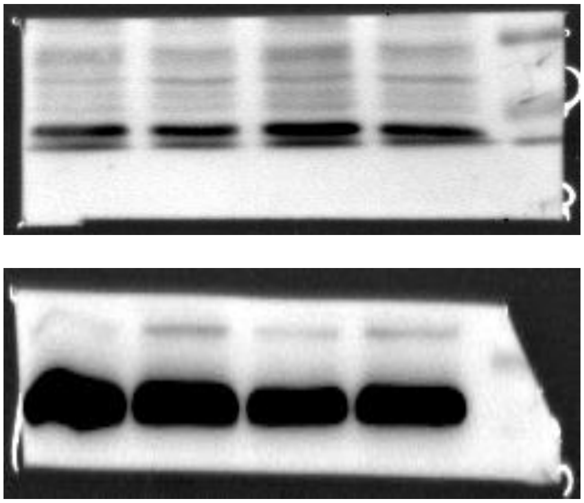

Figure 5. Western Blot Raw Images

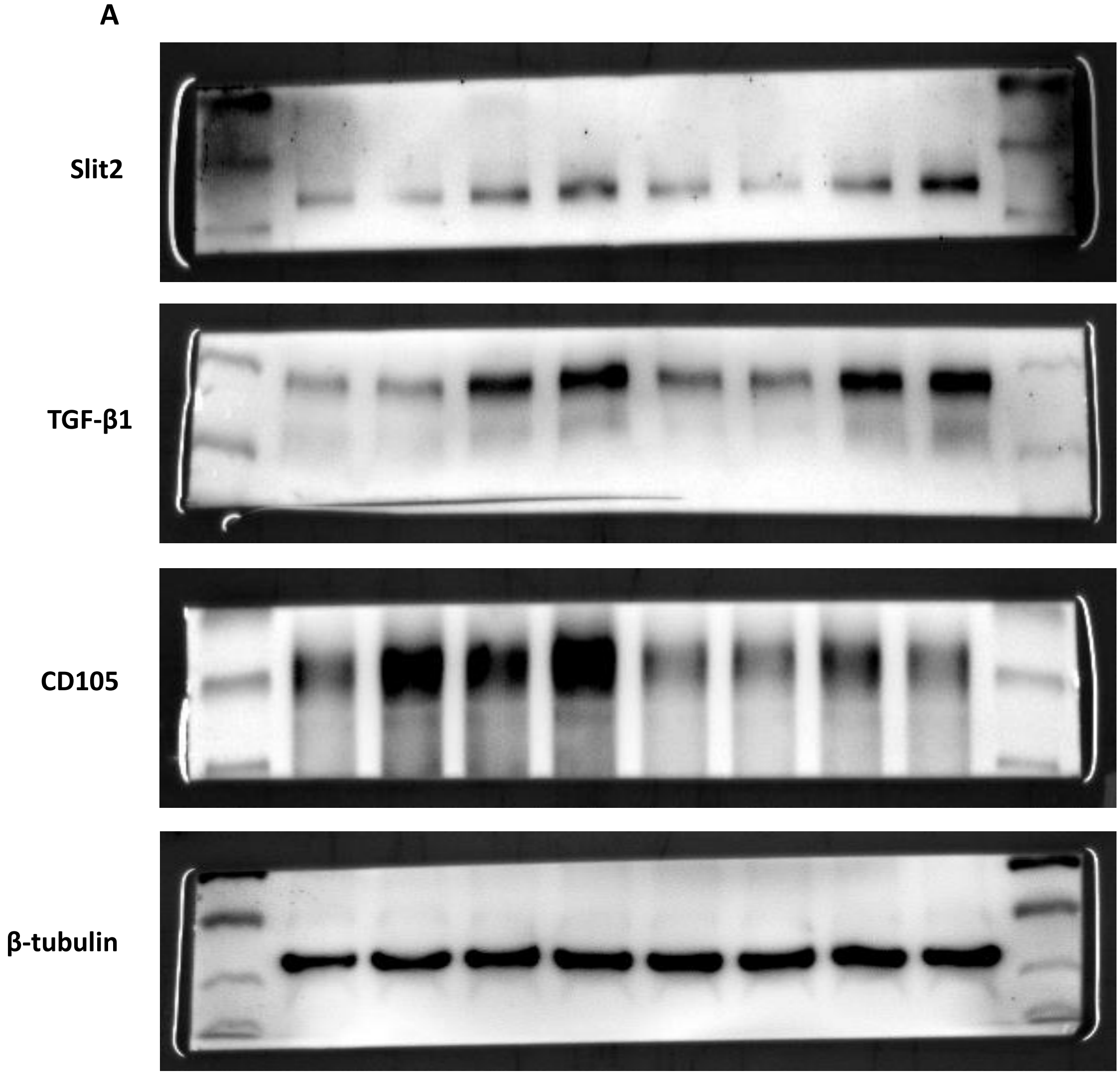

Supplement: Supplementary file 1 — WB raw data [file 41419_2024_6546_MOESM1_ESM.pdf]
